# A disposable biosensing system for analysis of CA125 in real human serum samples

**DOI:** 10.55730/1300-0527.3748

**Published:** 2025-06-10

**Authors:** Meltem AFŞAR, Berfin VURAL, Melike BİLGİ KAMAÇ, Mustafa Kemal SEZGİNTÜRK

**Affiliations:** 1 Department of Bioengineering, Faculty of Engineering, Çanakkale Onsekiz Mart University, Çanakkale, Turkiye; 2 Department of Chemistry, Faculty of Science, Çankırı Karatekin University, Çankırı, Turkiye

**Keywords:** CA125, biosensor, ovarian cancer, p-benzoquinone

## Abstract

Several biomarkers have been developed to track the development of ovarian cancer and identify the illness at an early stage. A promising development for ovarian cancer is using cancer antigen 125 (CA125). The objective of this work is to identify the CA125 marker by utilizing an indium tin oxide polyethylene electrode. The advantage of the designed biosensor is that it is very cheap, disposable, practical, and easy to use. The necessary parameters for the developed biosensor have been optimized in detail. Repeatability, reproducibility, regeneration, storage, and selectivity studies have been completed to characterize the proposed biosensor. Electrochemical impedance spectroscopy and cyclic voltammetry techniques were used for all experimental investigations of the proposed biosensor. The immunosensor offers a large linear detection range (0.01–100 pg/mL). Moreover, this developed sensor has a 0.018 pg/mL limit of detection and a 0.06 pg/mL limit of quantification. The high accuracy of this biosensor was observed in five commercial human serums.

## 1. Introduction

Cancer, defined as uncontrollable cell division and proliferation, is a highly complex disease under the influence of genetic and environmental conditions [1]. The most common cause of death from gynecologic cancer is ovarian cancer [2]. The high mortality rate is due to the late diagnosis of most cases. Measurement of the levels of cancer biomarkers is crucial for the early diagnosis of ovarian cancer [3]. Biomarkers have played a significant role in detecting and managing ovarian cancer. Many biomarkers for ovarian cancer have been the subject of substantial and in-depth research [4]. In recent years, CA125, also known as Carbohydrate Antigen 125, has played a significant role in ovarian cancer screening, diagnosis, and management [5]. The CA125 antigen is a mucin-like glycoprotein with a molecular weight of approximately 200,000 kDa [6]. The CA125 cancer biomarker is not very specific for ovarian cancer because it is also found in bladder, breast, lung, liver, and pancreatic cancer. However, CA125 levels are measured in routine screenings [7,8]. In 99% of healthy individuals, the serum level is below 35 U/mL, and in 99.7%, it is below 65 U/mL [9]. A concentration of 2.68 U/mL of CA125 protein is equivalent to 100 pg/mL.

It is important to produce biosensors with many advantages, such as sensitivity and practicality, for the early detection of CA125 protein. Biosensors are made from stable, cost-effective, durable, and electrochemically detectable materials for clinical use [10]. A biosensor combines a biological component with a target substance to produce an output (transducer) [11]. Electrochemical biosensors are analytical devices that combine the selectivity of biological recognition elements (such as enzymes or antibodies) with the sensitivity and specificity of electrochemical measurements. These biosensors detect biological analytes (such as glucose, cholesterol, or DNA) by converting a biochemical reaction into an electrical signal, which can then be measured and quantified [12,13].

Due to its unique features, such as low surface electrical resistance, physical stability, and simplicity of chemical modification, the indium tin oxide polyethylene (ITO-PET) electrode is a promising material widely employed in electrochemical biosensing applications [14–16]. The ITO-PET electrode served as the working electrode in the proposed biosensor. An electrochemical approach was employed to quantify the interaction between the antibody and the antigen.

p-benzoquinone is a chemical molecule utilized in biosensors due to its role as an electron acceptor. An electrochemical biosensor can be created by combining it with enzymes or other biological substances [17]. Benzoquinone-based biosensors are attractive because they can provide high sensitivity, selectivity, and stability. However, they also have some limitations, such as the need for relatively high concentrations of benzoquinone and the fact that other compounds in the sample can influence the electrochemical signal [18]. It is worth noting that there is still ongoing research to improve benzoquinone-based biosensors’ performance and find new applications [19]. This study used p-benzoquinone to immobilize CA125 on the ITO-PET surface.

In this research, the cyclic voltammetry (CV) and electrochemical impedance spectroscopy (EIS) methods were used to electrochemically follow the steps of making the CA125 immunosensor. A detailed optimization of several parameters (p-benzoquinone concentration and incubation time, antibody concentration, antibody, and antigen incubation time) was also performed.

The created biosensor exhibited high reproducibility and repeatability. This study examined the reusability of the ITO-PET-based electrode and determined that it enabled rapid regeneration rates. Finally, CA125 analysis was performed on commercially available human serum samples, and it was concluded that the biosensor produced accurate recovery values.

## 2. Materials and methods

### 2.1. Materials and instruments

The ‘Supporting Information’ section contains a list of the reagents, materials, and apparatus utilized in this research.

### 2.2. Electrochemical measurement

The ‘Supporting Information’ section contains information about the measurement.

### 2.3. Biosensor immobilization steps

**Figure 1:**
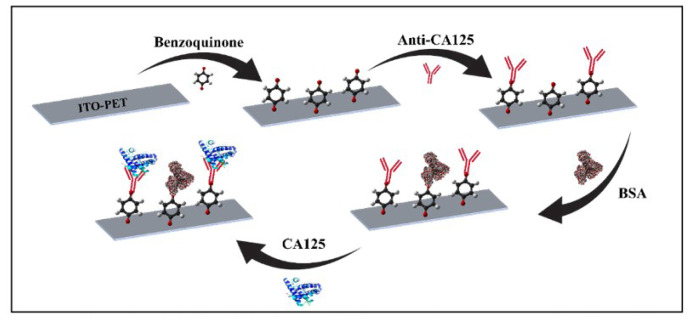
Illustration of the immobilization steps for the CA125 immunosensor on the p-benzoquinone-modified ITO-PET surface.

The ITO-PET layer was first cleaned using acetone, soap solution, and ultrapure water. The cleaning technique was performed using an ultrasonic bath for 10 min at each step. The layer was then exposed to a p-benzoquinone solution for 60 min. As a result, the binding of -OH groups to the ITO-PET surface was successfully achieved. To ensure the formation of a covalent bond between anti-CA125 and the surface, the electrode was immersed in a concentration of anti-CA125 solution for 45 min. Following immobilization of anti-CA125, active ends that did not bind to anti-CA125 were blocked using a bovine serum albumin (BSA) solution for 1 h. At each stage of immunosensor preparation, the electrode was gently rinsed with ultrapure water. Figure 1 shows the immobilization steps.

## 3. Results and discussion

### 3.1. Biosensor fabrication steps

**Figure 2:**
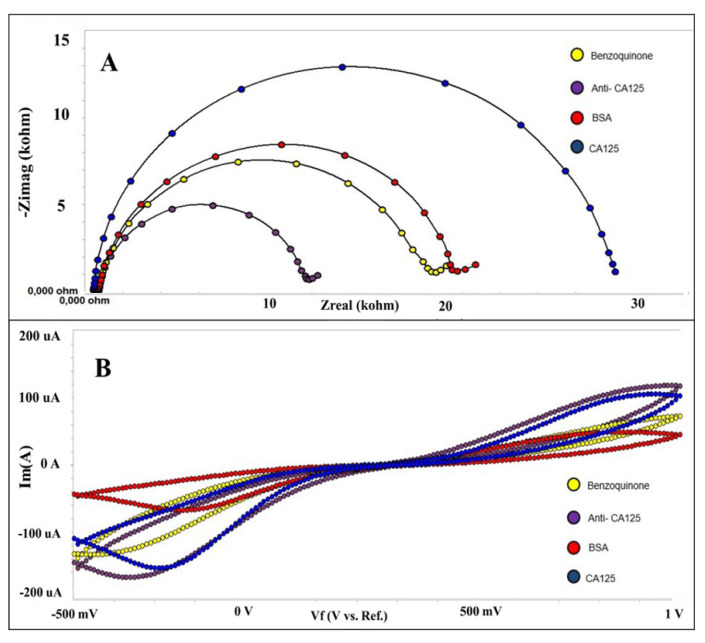
Immobilization steps of the biosensor [(A) Electrochemical impedance spectra obtained for the fabrication steps. (B) Cyclic voltammograms obtained for the fabrication steps].

The immobilization method is an important part of developing a biosensor that is sensitive, stable, and reliable. For this reason, the immobilization steps must be performed carefully and correctly. Electrochemical methods, including EIS and CV, were used to monitor the effect of the immobilization steps on the biosensor surface. Figures 2A and 2B present the EIS and CV results. Initially, the cleaned electrodes were treated with a p-benzoquinone solution. Thus, -OH groups were formed on the electrode surface, and benzoquinone interacted with reactive oxygen. Unpaired electrons in the oxygen atom are thought to electrostatically repel the negatively charged redox probe. As a result, there was a noticeable increase in charge transfer resistance. A significant decrease in the impedance value was observed after anti-CA125 immobilization. The amine terminals of the antibody are thought to contribute to conductivity. Consequently, anti-CA125 proteins facilitated the diffusion of the redox probe to the electrode surface. In the final immobilization step, the electrode surface was treated with a 0.5% BSA solution. Due to the blocking property of the BSA protein, the electrode surface became effectively insulating. Consequently, a considerable rise in the EIS signal was recorded due to the increased charge transfer resistance. In addition to measuring EIS at each immobilization phase, the CV approach was employed to validate these results. Cyclic voltammetry measurements supported the EIS results. As seen in Figure 2B, the immobilization of anti-CA125 proteins onto the electrode surface introduced an insulating layer which caused a decrease in the cathodic and anodic peak currents. Moreover, BSA application also affected the surface by decreasing the peak currents. Finally, the interaction between anti-CA-125 and anti-CA-125 increased the insulating layer on the electrode surface which resulted in more decrease in peak currents. All these CV results were in agreement with the electrochemical impedance spectroscopy.

### 3.2. Optimization studies

#### 3.2.1. Optimization of p-benzoquinone concentration

**Figure 3:**
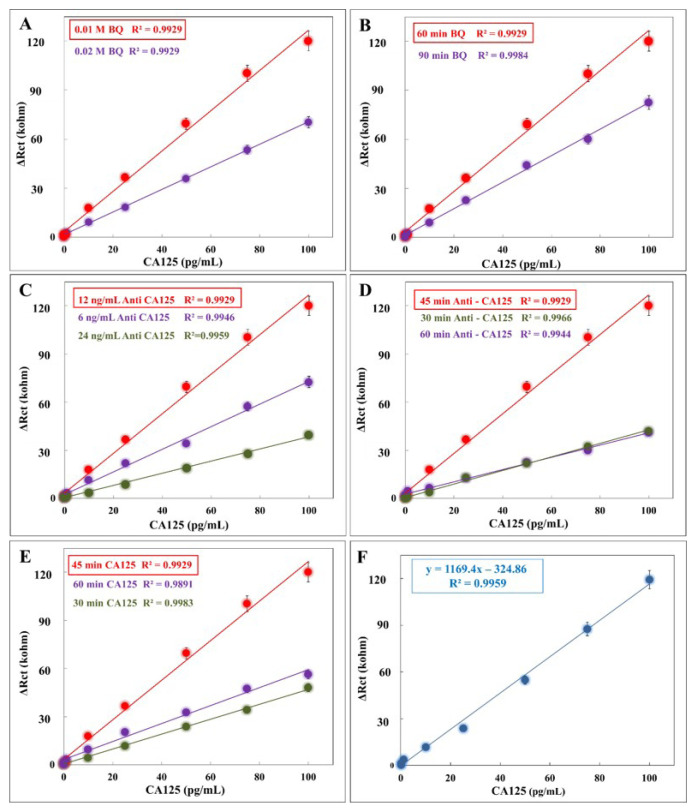
Optimization steps for the biosensor [(A) The effect of the p-benzoquinone concentration (BQ) on the biosensor performance. (B) The effect of the p-benzoquinone incubation duration on the biosensor performance (C) The effect of anti-CA125 concentration on the biosensor performance. (D) The effect of anti-CA125 incubation duration on the biosensor. (E) The effect of CA125 incubation duration on the biosensor. (F) Linear calibration graph for CA125].

The most important step in designing a biosensor is to perform optimization studies. Initially, the influence of p-benzoquinone concentration on biosensor performance was examined. Three concentrations—0.01 M, 0.02 M, and 0.005 M—were tested to optimize the p-benzoquinone concentration. The calibration graphs are shown in Figure 3A. No response was observed at 0.005 M benzoquinone concentration. This was interpreted to mean that the concentration did not enhance to the conductivity of ITO-PET. The maximum signal was measured when 0.01 M p-benzoquinone was utilized (Figure 3A). Based on this optimization, 0.01 M benzoquinone concentration was selected.

#### 3.2.2. Optimization of the immobilization time of p-benzoquinone

The second optimization parameter is the determination of the immobilization time of p-benzoquinone. In this study, three different parameters (30, 60, and 90 min) were studied. A response could not be obtained when benzoquinone incubation was tested at 30 min. It was thought that more time was needed for p-benzoquinone attachment. The highest signal for p-benzoquinone incubation was received at 60 min. As seen in Figure 3B, 60 min was selected for optimum incubation time.

#### 3.2.3. Optimization of anti-CA125 concentration

In the antibody concentration optimization study, 6 ng/mL, 12 ng/mL, and 24 ng/mL anti-CA125 concentrations were examined. Figure 3C depicts calibration curves for various anti-CA125 concentrations. Charge transfer resistance decreased at the highest antibody concentration (24 ng/mL). This is the result of surface deterioration. A high concentration of antibodies can damage the surface. The highest signal was obtained at 12 ng/mL anti-CA125 concentration. For this reason, a 12 ng/mL anti-CA125 concentration was chosen.

#### 3.2.4. Optimization of the incubation time of anti-CA125

In this anti-CA125 incubation time optimization study, three parameters (30, 45, and 60 min) were studied. The calibration curves obtained from the designed biosensor are compared in Figure 3D. The low signal observed during the 30-min incubation period indicates insufficient surface binding. During the 60-min incubation period, excessive anti-CA125 was considered to accumulate on the surface. Therefore, a low signal was obtained. The optimal incubation time for anti-CA125 was determined to be 45 min.

#### 3.2.5. Optimization of the incubation time of CA125

Finally, the interaction of the electrode surface with various CA125 incubation times was investigated. Trials were carried out at 30, 45, and 60 min. Figure 3E shows the calibration graphs for CA125. When the calibration curves were evaluated, the optimal time for interaction between CA125 and surface-immobilized anti-CA125 was 45 min. This result can be attributed to the negative effect of the high density of the electrode surface, as incubating the CA125 for more than 45 min significantly reduces the charge transfer resistances. Moreover, if CA125 is incubated for less than 45 min, CA125 proteins can be easily separated from the electrode surface without binding.

### 3.3. Analytical performance of the developed biosensor

After the optimization studies, the CA125 calibration graph for the biosensor with the optimal parameters was constructed. The findings of the characterization studies were derived using the equation obtained from the calibration graph. The calibration graph is shown in Figure 3F. The developed biosensor represents the linear range of CA125 from 0.01 pg/mL to 100 pg/mL. As can be understood from the linear detection range, the 10,000-fold linear detection range will enable the developed biosensor to be used in the analysis of samples with different concentrations in a wide range.

**Figure 4:**
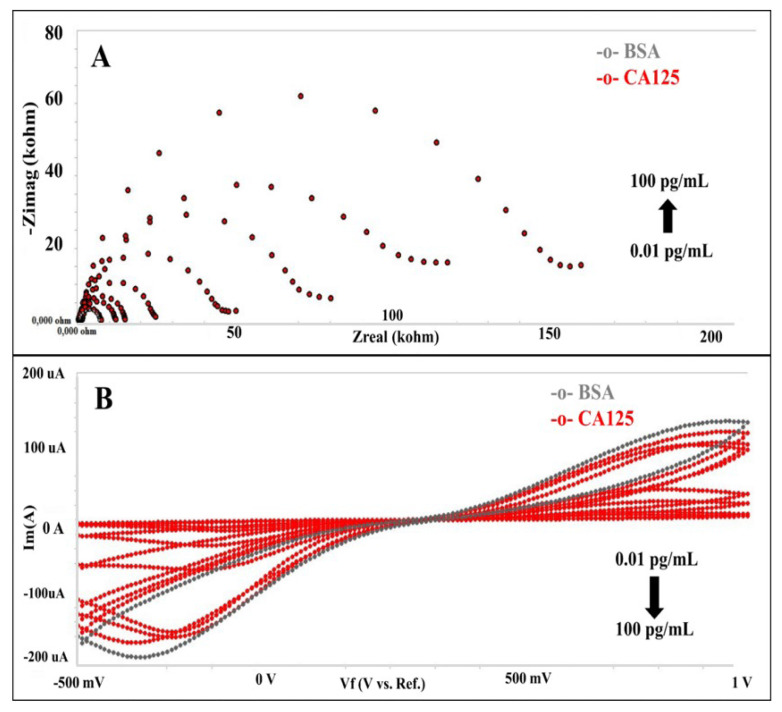
Electrochemical signals of the biosensor against CA-125 [(A) Electrochemical impedance spectra obtained for the different concentrations of CA-125. (B) Cyclic voltammograms obtained for the different concentrations of CA-125].

The biofunctionalized electrode surface was incubated with increasing concentrations of CA125. The changes in the electrochemical impedance spectra and cyclic voltammograms obtained with increasing concentrations of CA125 are shown in Figures 4A and 4B. The semicircle of the Nyquist graph increased in direct proportion to the increasing concentration. Similarly, cyclic voltammograms revealed that rising concentrations of CA125 decreased peak currents. The increase in impedance indicated the specific binding between antibodies and antigens on the electrode surface. Similarly to the EIS results, when the CA125 target concentration increased in the CV analysis, the peak currents decreased due to the formation of an insulating protein layer on the electrode.

#### 3.3.1. Repeatability and reproducibility

Repeatability and reproducibility are important characterization studies. To investigate the repeatability of the proposed biosensor, measurements were made at 50 pg/mL CA125 concentration with 20 independent electrodes under the same fabrication conditions. From these results, it was determined that the average CA125 concentration detected by the biosensor was 50.51 pg/mL, the relative standard deviation (RSD, %) was 14.41%, and the standard deviation was 7.28 pg/mL. These findings demonstrated a relatively acceptable repeatability of the ITO-based disposable biosensor even at relatively high concentrations of CA125.

**Figure 5:**
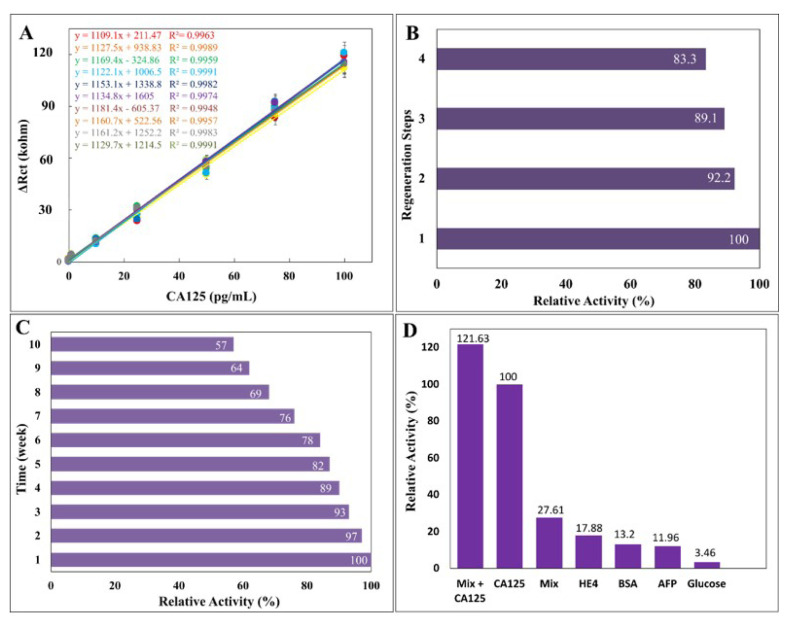
(A) Reproducibility of the biosensor. (B) Regeneration experiment results for the biosensor. (C) Storage stability of the biosensor. (D) Selectivity of the biosensor.

To assess the biosensor’s reproducibility, 10 different biosensor systems were prepared. How each biosensor system responded to the CA125 antigen at concentrations ranging from 0.01 pg/mL to 100 pg/mL was examined. Figure 5A shows the calibration graphs. The linearity of the graphs from the biosensors’ EIS curves shows a high degree of reproducibility.

#### 3.3.2.Regeneration study

Regarding surface stability and electrode cost, biosensor regeneration is an important attribute. Thus, regeneration research was conducted to assess the ITO-PET electrode’s surface stability. The ITO-PET electrode was prepared under ideal circumstances for biosensor regeneration capability. The electrode was subsequently treated with CA125 and 0.1% HCl solution. The dilute acid solution abolished the transient surface interactions between CA125 and anti-CA125. EIS measurements were performed following these procedures. The acid solution entirely damaged the electrode surface after four iterations of this operation. After the fifth incubation, there was no response from the biosensor. The biosensor retained nearly all its activity during the regeneration steps, with regeneration ending at 83.3% (Figure 5B).

#### 3.3.3. Stability of the biosensor

Determining the biosensor’s stability is highly dependent on its storage life. The biosensor’s stability provides information on its shelf-life. Every week, CA125 was incubated with biosensors made under ideal conditions. Ten different electrodes were prepared and stored at 4 °C in the dark. The electrodes were then incubated with CA125 at optimum biosensor parameters every week. Figure 5C shows the storage capacity of the biosensor. The biosensor showed an 11% activity loss by the end of the fourth week. The biosensor continued to work with an efficiency of 57% in the 10th week. In order to increase the storage stability of the biosensor, certain precautions can be taken both during the immobilization phase and during the storage period. For example, observing certain orientation processes during antibody immobilization can contribute to increasing storage stability. In addition, improving storage conditions by storing in sterile conditions and away from humidity also has the potential to positively affect storage stability.

#### 3.3.4. Biosensor selectivity experiments

The biosensor’s ability to selectively detect the target CA125 solution was tested to see if there were any nonspecific interactions. Seven different electrodes were prepared under optimum conditions. Each electrode was treated individually with a solution containing CA125 (75 pg/mL), HE4 (1 pg/mL), BSA (0.5%), AFP (5 ng/mL), and D-glucose solution (0.5 M). A mixed solution containing these five markers was prepared, and an electrode was incubated. In addition, another mixed solution without CA125 was prepared, and an electrode was incubated in this mixture. The results are shown in Figure 5D.

#### 3.3.5. Kramers-Kronig transform

The Kramers-Kronig transform was used to see whether outside factors affect the impedance spectra of the proposed CA125 biosensor system. The biosensor’s impedance curves overlapped at every stage of immobilization, which supported its stability and precision.

**Figure 6:**
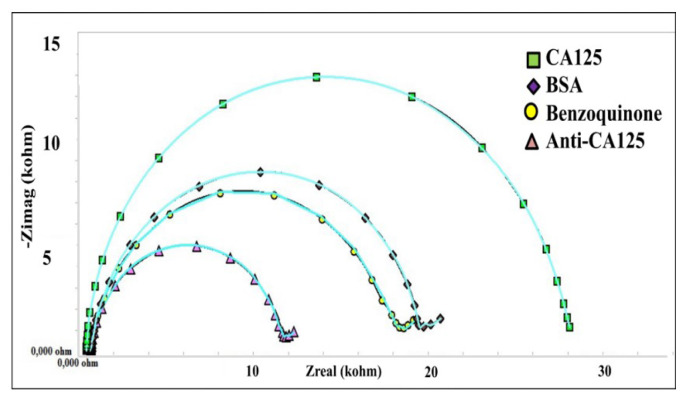
Kramers-Kronig transform of the designed biosensor.

In a Kramers-Kronig transform, if an impedance response that is obtained is not causal, linear, or stable, the spectrum obtained as a result of mathematical calculations does not exactly coincide with the spectrum obtained experimentally. The calculations we obtained in our studies are quite compatible, as seen in Figure 6.

### 3.4. Analysis of the CA125 in commercial human serum

This study demonstrated the designed biosensor’s usability for clinical applications. Before testing the usability of our biosensor in the analysis of real samples, it was deemed appropriate to compare biosensor systems that were developed in the past and used in real sample analyses. To date, many different kinds of biosensing systems have been reported. One of these was based on a dual analysis system developed by our team. In this system, both HE4 and CA125 could be determined simultaneously. However, it is understood that the new immobilization method used for anti-CA125 immobilization in this study improves the biosensor performance in terms of both the lower limit of detection and the linear determination range. Some CA125 biosensors are compared in Table 1.

In our system, the standard addition method was used in serum samples to determine the amount of CA125. The serum samples provided were diluted with phosphate buffer (50 mM, pH 7.0). EIS data were obtained by adding CA125 (0.05 pg/mL and 50 pg/mL) to the serum sample at different concentrations. The RSD and recovery percentages for each serum procedure were computed using the calibration graph equation and these data. Table 2 lists the conclusions of the calculations performed using serum samples. The findings demonstrate that the proposed biosensor can detect CA125 in human serum samples and that clinical adaptation is feasible.

## 4. Conclusion

CA125 is often used as a tumor marker to monitor ovarian cancer progression. Elevated concentrations of CA125 in the bloodstream can serve as an indication of cancer. However, it can also be attributed to other illnesses such as endometriosis, uterine issues, and pelvic inflammatory disease. Therefore, CA125 is not a definitive diagnostic test for ovarian cancer but a valuable tool for monitoring the disease and assessing response to treatment. This work presents the fabrication of a disposable, low-cost, reusable, sensitive, and reproducible CA125 immunosensor with ITO-PET electrodes. A highly sensitive biosensor was produced with an operating range of 10,000-fold (0.01–100 pg/mL). The immobilization of the developed biosensor consists of four basic steps (BQ, Anti-CA125, BSA, and CA125). What distinguishes this study is the use of p-benzoquinone, as it is rarely employed in biosensor applications. Characterization studies confirmed that this immunosensor exhibits good analytical performance. In addition, the proposed biosensor successfully detected CA125 concentrations in serum samples. This biosensor has many advantages for future clinical applications and point-of-care testing. It is considered that the immobilization procedure performed on the ITO surface may have a high potential for routine clinical applications. For this purpose, turning the ITO surface into a screen-printed electrode that can be integrated with portable systems is essential for future applications. In addition, a portable electrochemical analysis system for clinical applications can be developed by optimizing and developing miniaturized analysis systems for this purpose in the near future.

## Supporting information

### Materials and instruments

The biorecognition materials (CA125 antibody, CA125 antigen, and 0.5% BSA (bovine serum albumin) protein), as well as the chemicals (p-benzoquinone and ethanol) and a commercial serum sample, were all purchased from Sigma-Aldrich (St. Louis, M.O., USA). This study used an Ag/AgCl (saturated with KCl) reference electrode and a platinum wire counter electrode. The reference and counter electrodes were provided by BASi (West Lafayette, IN, USA). A phosphate buffer (50 mM, pH 7.0) was used to create whole proteins, kept at 20 °C until required. Phosphate buffer at pH 8.0 was used to prepare the p-benzoquinone solution. ITO-PET films (surface permeability: 550 nm (>79%), resistance: 60 Ω/square) taken from sigma were used as the working electrode. All electrochemical impedance and cyclic voltammetry were performed throughout the investigation using an EChem Analyst-equipped Gamry Potentiostat/Galvanostat (Reference 600, Gamry Instruments, Warminster, PA, USA). A PURELAB flex 3 & 4 ultrapure water purification system (ELGA LC 134 model, ELGA LabWater, High Wycombe, UK) was used during all preparation stages of the proposed immunosensor for ultrapure water.

### Electrochemical measurement

Electrochemical experiments of the CA125 immunosensor were performed by cyclic voltammetry and electrochemical impedance spectroscopy. The CV was subjected to measurements in a redox solution containing 0.1 M KCl and 5 mM K3[Fe(CN)6]/K4[Fe(CN)6] with a step size of 10 mV and a scanning speed of 100 mV/s (1:1). Electrochemical impedance measurements in the redox solution were taken using a 10 mV alternating current. The range of the impedance is 10,000 to 0.05 Hz. All measurements were made in the reaction cell at 25 °C.

## Figures and Tables

**Table 1:** Comparison of various CA125 biosensors.

Method	Immobilization strategy	LOD	Linear range	Ref.
Amperometry	Sandwich-type immunosensor	77 fg/mL	0.2–1000 pg/mL	[20]
Amperometry	Anti CA125 – GHM – PTH – GCE immunosensor	1.3 U/mL	4.5–36.5 U/mL	[21]
Voltammetry	Cys-AuNPs – ERGO-P (DA)	0.1 U/mL	0.1–400 U/mL	[22]
Electrochemical impedance spectroscopy	Immunosensor using AuNPs screen-printed electrode	6.7 U/mL	0–100 U/mL	[23]
Electrochemical impedance spectroscopy	Micro-flow label-free biosensor	7 U/mL	0–100 U/mL	[24]
Fluorescence spectroscopy	CA125 aptamer-directed AgNCs assay	1.26 ng/mL	2ng/mL–6.7 μg/mL	[25]
Voltammetry	Anti CA125/MOF-808/CNT	0.0005 ng/mL	0.001–30 ng/mL	[26]
Impedimetry	Anti-CA125/RGO/PTH/AuNP/	1.17 pg/mL^−1^	1–100 pg mL^−1^	[27]
Impedimetry	Anti-CA125/p-BQ	0.018 pg/mL	0.01–100 pg/mL	This work

LOD: Limit of detection.

**Table 2:** Results of calculations using serum samples.

Serum samples	Measured CA125 (pg/mL)	Spiked CA125 (pg/mL)	Total measured CA125 (pg/mL) (n=3)	RSD (%) (n=3)	Recovery (%)
1	0.959	0.05 50	0.994/1.008/1.021 56.982/46.775/53.862	0.139 3.100	99.861 103.100
2	2.428	0.05 50	2.475/2.482/2.489 49.798/50.268/53.898	0.161 2.111	100.161 97.889
3	2.933	0.05 50	2.986/2.979/2.992 56.786/52.291/55.087	0.067 3.377	100.067 103.377
4	1.754	0.05 50	1.805/1.794/1.810 52.156/49.619/55.397	0.055 1.228	99.945 101.228
5	0.637	0.05 50	0.698/0.684/0.694 53.784/52.321/48.876	0.727 2.020	100.727 102.020
